# Using Drains to Prevent Seroma Formation after Breast Surgery

**DOI:** 10.1097/GOX.0000000000002504

**Published:** 2019-11-19

**Authors:** Leo D. Farrell

**Affiliations:** From Farrell Plastic Surgery & Laser Center, Mechanicsburg, Penn.

## Sir,

My recent experience with circumvertical breast reduction patients who have developed complications from seroma have made me reconsider using drains. Of my last 10 patients, 2 required aspiration of seromas of ~50 ml because of persistent swelling and firmness. Another patient developed an infected seroma of ~100 ml, which required exploration, drainage, and antibiotics 5 weeks after surgery. I have noticed that patients have significantly more swelling and firmness when drains are not used. There are multiple studies in the literature which have demonstrated that postoperative use of drains has led to no difference in hematoma rates, wound healing complications, or greater patient discomfort.^1–4^ However, Anzarut et al^5^ have found an increased risk of postoperative drainage with the superior pedicle breast reduction technique. Their patients were found to have on average 83 ml more drainage during the first 24 hours postoperatively. The problems related to using drains have been much easier to manage than complications of seroma formation in my patients.
